# Clinically Relevant *Escherichia* *coli* Isolates from Process Waters and Wastewater of Poultry and Pig Slaughterhouses in Germany

**DOI:** 10.3390/microorganisms9040698

**Published:** 2021-03-28

**Authors:** Mykhailo Savin, Gabriele Bierbaum, Judith Kreyenschmidt, Ricarda Maria Schmithausen, Esther Sib, Silvia Schmoger, Annemarie Käsbohrer, Jens Andre Hammerl

**Affiliations:** 1 Institute of Animal Sciences, University of Bonn, 53113 Bonn, Germany; j.kreyenschmidt@uni-bonn.de; 2 Institute for Hygiene and Public Health, Medical Faculty, University of Bonn, 53113 Bonn, Germany; ricarda.schmithausen@ukbonn.de (R.M.S.); esther.sib@ukbonn.de (E.S.); 3 Institute for Medical Microbiology, Immunology and Parasitology, Medical Faculty, University of Bonn, 53113 Bonn, Germany; g.bierbaum@uni-bonn.de; 4 Department of Fresh Produce Logistics, Hochschule Geisenheim University, 65366 Geisenheim, Germany; 5 Department for Biological Safety, German Federal Institute for Risk Assessment, 10589 Berlin, Germany; silvia.schmoger@bfr.bund.de (S.S.); annemarie.kaesbohrer@bfr.bund.de (A.K.); 6 Department for Farm Animals and Veterinary Public Health and Epidemiology, Unit of Veterinary Public Health and Epidemiology, University of Veterinary Medicine Vienna, 1210 Vienna, Austria

**Keywords:** *E. coli*, ExPEC, UPEC, virulence, MDR, resistance, slaughterhouse, wastewater

## Abstract

*Escherichia coli* is frequently associated with multiple antimicrobial resistances and a major cause of bacterial extraintestinal infections in livestock and humans. However, data on the epidemiology of (i) multidrug-resistant (MDR) and (ii) extraintestinal pathogenic *E. coli* (ExPEC) in poultry and pig slaughterhouses in Germany is currently lacking. Selected *E. coli* isolates (*n* = 71) with phenotypic resistance to cephalosporins from two poultry and two pig slaughterhouses expressing high MDR rates (combined resistance to piperacillin, cefotaxime and/or ceftazidime, and ciprofloxacin) of 51.4% and 58.3%, respectively, were analyzed by whole-genome sequencing. They constituted a reservoir for 53 different antimicrobial resistance determinants and were assigned various sequence types, including high-risk clones involved in human infections worldwide. An ExPEC pathotype was detected in 17.1% and 5.6% of the isolates from poultry and pig slaughterhouses, respectively. Worryingly, they were recovered from scalding water and eviscerators, indicating an increased risk for cross-contaminations. Uropathogenic *E. coli* (UPEC) were detected in the effluent of an in-house wastewater treatment plant (WWTP) of a poultry slaughterhouse, facilitating their further dissemination into surface waters. Our study provides important information on the molecular characteristics of (i) MDR, as well as (ii) ExPEC and UPEC regarding their clonal structure, antimicrobial resistance and virulence factors. Based on their clinical importance and pathogenic potential, the risk of slaughterhouse employees’ exposure cannot be ruled out. Through cross-contamination, these MDR *E. coli* pathotypes may be introduced into the food chain. Moreover, inadequate wastewater treatment may contribute to the dissemination of UPEC into surface waters, as shown for other WWTPs.

## 1. Introduction

The use of antimicrobials for veterinary purposes in Germany decreased over the years, encouraged by the nationwide antibiotics minimization concept in animal keeping. Between 2011 and 2017, a decrease of 57% (~973 tons) was observed in sales data. However, from 2015 onward, sold quantities of highest priority critically important antimicrobials (HPCIA, i.e., 3rd generation cephalosporins, fluoroquinolones, macrolides, polypeptides), which are particularly important for public health, stagnated or even slightly increased in the case of fluoroquinolones and polypeptides [1].

Because of the effects of cross-resistance and horizontal gene transfer, the consumption of antimicrobials belonging to HPCIA classes in veterinary medicine may contribute to the development, selection, and proliferation of resistance to clinically relevant antimicrobials [2,3]. Important examples are *Enterobacteriaceae* from the poultry and pig production chains that produce extended-spectrum β-lactamases (ESBLs). They are resistant to piperacillin and fluoroquinolones (3MDRO, multidrug-resistant Gram-negative organisms exhibiting resistance to three antibiotic groups ureidopenicillins, third/fourth-generation cephalosporins and fluoroquinolones) [4,5,6,7]. Furthermore, *Enterobacteriaceae* carrying pAmpC, *bla*_OXA-1_ or with (hyper)production of inhibitor-resistant *bla*_TEM_-variants, the occurrence of which is mostly reported for human clinical isolates, compromise the activity of β-lactam/β-lactamase inhibitor combinations (e.g., ceftolozane-tazobactam (C/T) and/or ceftazidime-avibactam (CZA)) [8,9].

Antimicrobial resistance has adverse consequences for human, animal, and environmental health (“One Health”). It was estimated that if no further action is taken, an annual death rate of 10 million humans may be reached by 2050 due to infections with multidrug-resistant (MDR) bacteria [10]. *Escherichia coli* is often associated with multiple antimicrobial resistance and is one of the major causes of bacterial extraintestinal infections in humans, i.e., urinary tract infections (UTIs), infections of the skin and soft tissues, respiratory tract, meningitis as well as pneumonia [11]. In the poultry production system, pathogenic *E. coli* is also responsible for extraintestinal infections, collectively called colibacillosis, that are associated with the enormous economic loss [12]. Such strains belong to avian pathogenic *E. coli* (APEC). *Escherichia coli* strains responsible for extraintestinal diseases in humans are termed extraintestinal pathogenic *E. coli* (ExPEC). APEC and ExPEC can harbor diverse virulence factors, including adhesins, toxins, iron uptake systems (siderophores) and capsules, which are necessary for colonization and infection of different physiological compartments and often overlap [13]. Thus, some APEC strains are host independent and can cause diseases in hosts other than poultry, such as pigs and humans, that underline their zoonotic potential [13,14].

Poultry and pigs carrying *E. coli* strains capable of causing extraintestinal infections and expressing the 3MDRO resistance phenotype introduce such bacteria into slaughterhouses. During subsequent processing, many process waters and wastewater arise that are contaminated by numerous bacteria, which mainly originate from livestock feces. This poses an elevated risk of colonization and infection of employees with occupational exposure to contaminated waters [15]. Furthermore, the introduction of clinically-relevant antibiotic-resistant bacteria into the food chain through cross-contaminations with polluted scalding water or process water from eviscerators cannot be ruled out [16]. Moreover, through inadequate wastewater treatment in in-house wastewater treatment plants (WWTPs), such bacteria could be discharged into the surface water and be further disseminated in the environment [17].

However, only limited data are available on the epidemiology of multidrug-resistant and extraintestinal pathogenic *E. coli* in German poultry and pig slaughterhouses. Thus, this study aims to characterize selected *E. coli* isolates expressing clinically relevant resistance phenotypes (3MDRO, ceftolozane-tazobactam (C/T) and/or ceftazidime-avibactam (CZA)), and which were allocated to the ExPEC groups regarding their antimicrobial resistance genes, population structure and virulence factors.

## 2. Materials and Methods

### 2.1. Sampling Sites and Sample Preparation

Collection and preparation of process waters and wastewater from two poultry and two pig slaughterhouses, as well as their in-house WWTPs, were conducted as previously described (Savin et al. 2020a, 2020b). Therefore, individual water samples (1 L) were collected using sterile Nalgene^®^ wide mouth environmental sample bottles (Thermo Fisher Scientific, Waltham, MA, USA). Afterward, they were transported to the laboratory in a Styrofoam box cooled to 5 ± 2 °C, manually filtered using stomacher strainer bags with a tissue filter (pore size, 0.5 mm; VWR, Radnor, PA, USA) and subjected to cultivation within 24 h after sampling. The investigated slaughterhouses were located in different federal states and were at least 100 km apart. Further information on selected slaughterhouses characteristics, sampling sites, number of samples taken at each sampling site and sampling dates is summarized in Tables S1–S3.

A total of 149 water samples were included in the present study. Briefly, 82 water samples originating from poultry slaughterhouses were taken from eight sampling sites: transport trucks (*n* = 5), transport crates (*n* = 10), stunning facilities (*n* = 10), scalders (*n* = 10), eviscerators (*n* = 10), aggregate wastewater from production facilities (*n* = 5) as well as influent (*n* = 16) and effluent (*n* = 16) of the in-house WWTPs) [6]. Another set of 67 water samples from pig slaughterhouses was collected in the delivery (transport trucks, *n* = 10; holding pens, *n* = 7) and unclean areas (scalding and dehairing water, *n* = 10; aggregate wastewater from production facilities, *n* = 10) as well as in the in-house WWTPs (influent (*n* = 15) and effluent (*n* = 15)) [7].

### 2.2. Isolation and Identification of Target E. coli

Isolation of ESBL-producing *E. coli* was conducted using CHROMagar ESBL selective media (Mast Diagnostica, Reinfeld, Germany) as previously described [6,7]. Isolates were purified by streaking out individual colonies on Columbia Agar supplemented with 5% sheep blood (ColSB, Mast Diagnostics, Reinfeld, Germany). Species identification was conducted using a MALDI-TOF MS employing a VITEK^®^ mass spectrometer (bioMérieux, Marcy-l´Étoile, France) equipped with the Myla™ software.

### 2.3. Antimicrobial Susceptibility Testing (AST) and Molecular Typing

AST was performed by broth microdilution using commercial screening system Micronaut S MDR MRGN (MERLIN, Gesellschaft für mikrobiologische Diagnostika GmbH, Bornheim-Hersel, Germany) and applying clinical cut-off values (EUCAST, version 9.0) as previously described [6]. This screening system was chosen in order to assess the clinical relevance of isolates recovered from slaughterhouses for human medicine. Multidrug-resistance phenotype (3MDRO) was defined as a combined resistance to piperacillin (PIP), cefotaxime (CTX) and/or ceftazidime (CAZ), and ciprofloxacin (CIP) [18]. The following antimicrobial agents were used in this study: temocillin, TEM; piperacillin-tazobactam, TZP; cefotaxime, CTX; ceftazidime, CAZ; ceftazidime-avibactam, CZA; ceftolozane-tazobactam, C/T; imipenem, IMP; meropenem, MEM; amikacin, AMK; ciprofloxacin, CIP; levofloxacin, LVX; sulfamethoxazole-trimethoprim, SXT; fosfomycin, FOF; colistin, CST.

For the determination of phylogenetic groups (A, B1, B2, C, D, E, F and clade I) and identification of extraintestinal pathogenic (ExPEC) strains, *E. coli* isolates were genotyped according to the method of Clermont and colleagues (2013) [19].

Allocation to ExPEC and uropathogenic *E. coli* (UPEC) pathotypes was done according to Johnson and colleagues (2003) [20] and Spurbeck and colleagues (2012) [21], respectively. The ExPEC pathotype was defined by the presence of ≥2 virulence genes (VGs) coding for adhesins (*papA*/*papC*, *sfa*/*foc*, *afa*/*dra*), protectins (*kpsM II*) and/or iron uptake systems (*iutA*). The presence of ≥2 VGs encoding iron uptake systems (*chuA*, *fyuA*), toxins (*vat*) and/or adhesins (*yfcV*) defined the UPEC pathotype.

### 2.4. DNA Preparation, Whole-Genome Sequencing and Bioinformatics Analysis

For paired-end, short-read whole-genome sequencing (WGS) on an Illumina NextSeq 500 device, DNA libraries prepared with the Nextera DNA Flex library preparation kit (Illumina^®^, San Diego, CA, USA) according to the manufacturer’s protocol were used. NextSeq sequencing was performed in 2 × 151 cycles with the Illumina^®^ NextSeq™ 500/550 Mid Output Kit v2.5 (300 cycles) (Illumina^®^, San Diego, CA, USA) (Borowiak et al. 2017). Isolation of genomic DNA for 1 mL liquid cultures of bacteria was conducted using the PureLink Genomic DNA Preparation Mini Kit (Invitrogen GmbH, Darmstadt, Germany) recommended by the manufacturers. Illumina raw reads were trimmed and subjected to de novo assembling with the Aquamis pipeline (https://gitlab.com/bfr_bioinformatics/AQUAMIS/, access date: September 2020) using fastp [22] for trimming and shovill (https://github.com/tseemann/shovill, access date: September 2020) for genome assembly. The Aquamis pipeline further uses mash v 2.1 for reference search [23] and quast v 5.0.2 for assembly quality control [24]. In silico based characterization of genetic features was performed with the Bakcharak pipeline (https://gitlab.com/bfr_bioinformatics/bakcharak, access date: September 2020) implementing ABRicate (https://github.com/tseemann/abricate, access date: September 2020) for antimicrobial/biocide resistance [25] and virulence factors detection [26]. Multi-locus sequence types (MLST) were predicted using mlst (https://github.com/tseemann/mlst, access date: September 2020) [27].

## 3. Results

### 3.1. Isolation and Selection of target E. coli

In general, out of 376 recovered *E. coli* isolates (*n* = 186 poultry slaughterhouses; *n* = 190 pig slaughterhouses), 71 isolates (18.9%) were chosen for further analysis. The selection was based on at least one of the following criteria: (i) development of a 3MDRO resistance phenotype; (ii) resistance to the newly approved drug combinations ceftazidime-avibactam and/or ceftolozane-tazobactam, (iii) allocation to the ExPEC phylogroups B2/D/F.

Of the selected *E. coli* isolates, 35 were recovered from process waters and wastewater generated in poultry slaughterhouses during operation and cleaning of facilities: transport trucks (*n* = 2); transport cages (*n* = 3); stunning facilities (*n* = 7); scalding water (*n* = 2); eviscerators (*n* = 6); aggregate wastewater from production facilities (*n* = 3); influent in-house WWTP (*n* = 5) and effluent in-house WWTP (*n* = 7). From process waters and wastewater accruing in pig slaughterhouses, 36 *E. coli* isolates were chosen: transport trucks (*n* = 4); holding pens (*n* = 3); scalding water (*n* = 3); production facilities (*n* = 7); influent biological WWTP (*n* = 9); influent chemical–physical WWTP (*n* = 4) and effluent biological WWTP (*n* = 6). Information on the isolation sites of individual isolates and their accession numbers are provided in Table S4.

### 3.2. Phenotypic Antimicrobial Resistance

**Figure 1:**
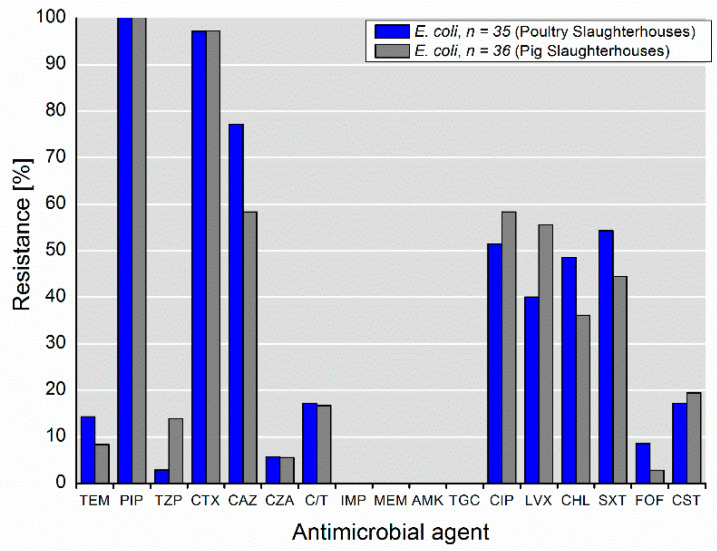
Phenotypical resistance to antimicrobial agents detected among selected *E. coli* isolates (*n* = 71) from wastewater and process water from poultry and pig slaughterhouses.

Selected *E. coli* isolates (*n* = 35 poultry slaughterhouses; *n* = 36 pig slaughterhouses) exhibited diverse resistance phenotypes, including resistance to highly and critically important antimicrobials for humans (Figure 1). Furthermore, they expressed high multidrug-resistance rates. Of the isolates recovered from the poultry and pig slaughterhouses, 51.4% and 58.3%, respectively, expressed combined resistance to piperacillin (PIP), cefotaxime (CTX) and/or ceftazidime (CAZ), and ciprofloxacin (CIP). The rates of resistance to combinations of β-lactam/β-lactamase inhibitor (i.e., piperacillin-tazobactam, TZP; ceftazidime-avibactam, CZA; ceftolozane-tazobactam, C/T) was between 2.9% and 17.1%, whereas the highest rates were observed for C/T. Furthermore, of the isolates recovered from the poultry and pig slaughterhouses, 17.1% and 19.4%, respectively, expressed resistance to colistin. Noteworthy, all isolates were susceptible to carbapenems (imipenem, meropenem), amikacin and tigecycline.

### 3.3. Characterization of Antimicrobial Resistance Genes

**Figure 2:**
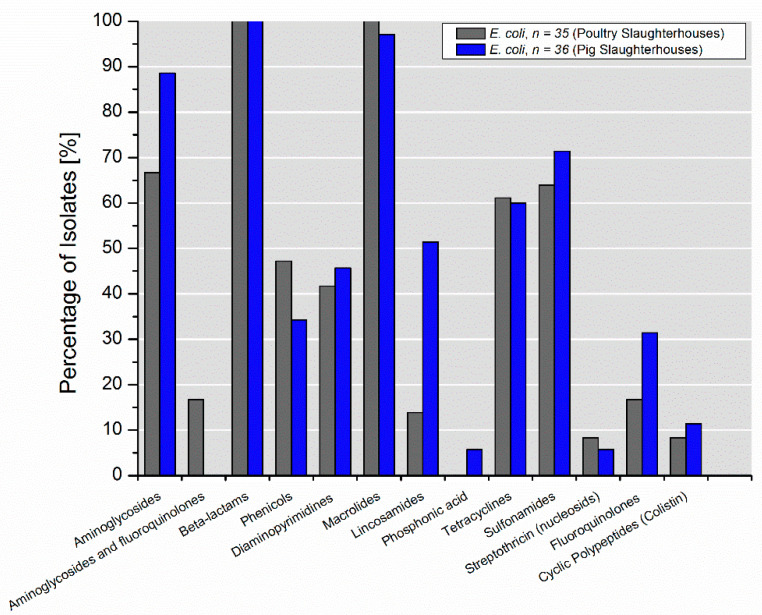
Percentage of selected *E. coli* isolates (*n* = 71) recovered from wastewater and process waters from poultry and pig slaughterhouses carrying genes mediating resistance to the specific classes of antimicrobials.

**Figure 3:**
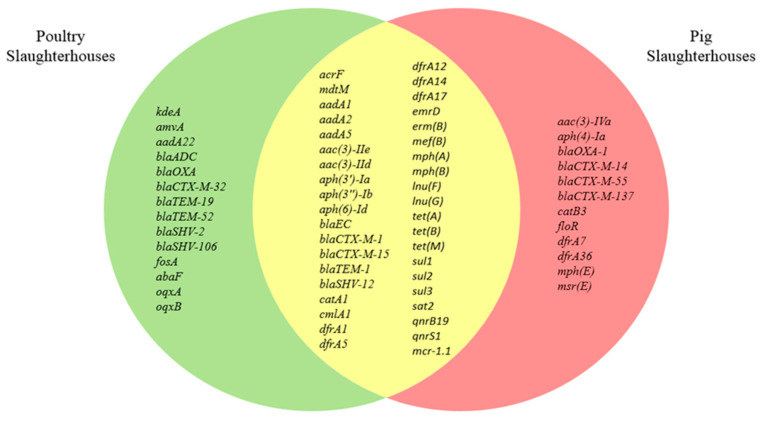
Antibiotic resistance genes identified in selected *E. coli* isolates (*n* = 71) from poultry and pig slaughterhouses.

In selected *E. coli* isolates from poultry slaughterhouses, 53 antimicrobial resistance genes (ARGs) belonging to 13 different classes were identified, whereas isolates from pig slaughterhouses represented a reservoir for 52 ARGs of 13 different classes. Of these, 39 ARGs were common for isolates from both poultry and pig slaughterhouses (Figure 2, Figure 3).

Compared to the isolates from pig slaughterhouses, a higher abundance of ARGs coding for resistance to lincosamides *lnu(F)* and *lnu(G)* (51.4% vs. 13.9%) was detected in isolates from poultry slaughterhouses (Table 1). Interestingly, 16.7% (6/36) of the isolates from pig slaughterhouses harbored gene encoding resistance to aminoglycosides and fluoroquinolones (*aac(6’)-Ib-cr5*). As expected, β-lactamases genes that belonged to the *bla*_ADC_, *bla*_OXA_, *bl*_EC_, *bla*_CTX-M_, *bla*_TEM_, *bla*_SHV_ families were detected in all selected isolates since selective media were used for their culturing. However, only five β-lactamases genes (*bl*_EC_, *bla*_CTX-M-1_, *bla*_CTX-M-15_, *bla*_TEM-1_, *bla*_SHV-12_) of 16 identified ones were present in isolates from both poultry and pig slaughterhouses. Interestingly, *bla*_-OXA-1_ was detected in combination with extended-spectrum β-lactamases (ESBLs), such as *bla*_CTX-M-1_ and *bla*_CTX-M-15_ only in isolates from both pig slaughterhouses. Noteworthy, ARGs coding for resistance to macrolides was detected in all isolates with very few exceptions among isolates from pig slaughterhouses. They were mostly represented by *emrD* or combinations of *emrD* with *mph(A)* or *mph(B)*. ARGs conferring resistance to phosphonic acid (*fosA, abaF*) and streptothricin (*sat2*) were found only rarely with abundances <10%.

In general, there was a good concordance between the resistance phenotypes and the resistance genes identified by WGS for isolates from both poultry and pig slaughterhouses.

### 3.4. Distribution of Phylogenetic Groups and MLST Sequence Types

Selected *E. coli* isolates from poultry slaughterhouses (*n* = 35) mainly belonged to the group B1 (34.3%), followed by F (17.1%), E (14.3%), A and D (each 11.4%), C (8.6%) as well as B2 (2.9%). Most of the isolates from pig slaughterhouses (*n* = 36) were also assigned to B1 group (41.7%), followed by A (25.0%), C (22.2%), B2 (5.6%) and D (5.6%).

MLST revealed a high genetic diversity of selected *E. coli* isolates. Overall, 66 isolates were assigned to 42 distinct previously described sequence types (STs), whereas five isolates exhibited novel STs (Table 2). Isolates from poultry slaughterhouses belonged to 25 different STs, whereas isolates from pig slaughterhouses exhibited 22 STs. Interestingly, five STs (ST10, ST58, ST101, ST117, ST224) were common for isolates from poultry and pig slaughterhouses.

### 3.5. Characterization of Virulence Genes

The virulence genes of the analyzed *E. coli* isolates are summarized in Table 3. In general, genes coding for virulence factors, such as adhesins, toxins, siderophores and capsules, were detected. Interestingly, almost all isolates carried *fimH* that codes for the adhesin on type 1 pili. Of note, 40.0% of isolates from poultry slaughterhouses (14/35) carried virulence gene *astA* encoding the enteroaggregative *E. coli* heat-stable enterotoxin (EAST1), whereas this virulence genotype was less present among isolates from pig slaughterhouses (16.7%, 6/36). Nevertheless, one isolate recovered from the wastewater used for cleaning pig transport trucks carried virulence determinants coding for α-hemolysin (*hlyD*) and cytotoxic necrotizing factor (*cnf1*). Furthermore, a high percentage of isolates was positive for different siderophores, such as aerobactin (*iutA*), salmochelin (*iroN*) and yersiniabactin (*fyuA*), whereas aerobactin was the most prevalent one. Noteworthy, the percentage of isolates carrying genes for group 2 capsule (*kpsM II*) was higher among isolates from poultry slaughterhouses (22.9%, 8/35) compared to those from pig slaughterhouses (5.6%, 2/36).

Of the isolates from poultry slaughterhouses, 17.1% (6/35) were assigned to ExPEC pathotype (*iutA*, *kpsM II*), isolated from scalding water, eviscerators and aggregate wastewater from production facilities. Interestingly, they mostly belonged to phylogroup F (3/6), followed by E (2/6) and B2 (1/6). Among the isolates from pig slaughterhouses, the abundance of ExPEC pathotype was lower. Of the selected *E. coli* isolates, 5.6% (2/36) belonged to ExPEC (phylogroup B2) carrying *sfa* (S fimbriae), *kpsM II* and *iutA*, *kpsM II*, respectively. They were isolated from wastewater used for cleaning of transport trucks and influent of biological WWTP. One isolate (D, ST117) recovered from the effluent of in-house WWTP of a poultry slaughterhouse (2.8%) carried *fyuA* and *vat* (vacuolating toxin), which define UPEC (uropathogenic *E. coli*) pathotype. UPEC (B2, ST1170) was also detected in wastewater used for cleaning pig transport trucks (2.8%, 1/36).

### 3.6. Heavy Metal and Biocide Resistance

The occurrence of heavy metal resistance genes in the analyzed *E. coli* isolates is shown in Table 4. In general, percentages of isolates carrying determinants conferring resistance to heavy metals were higher among isolates from pig slaughterhouses. They exhibited higher rates of resistance to copper, copper/silver, mercury, and silver. All isolates, but one from poultry slaughterhouses, carried genes conferring resistance to arsenic.

Of the isolates from poultry slaughterhouses, 42.9% (15/35) carried genes (*emrE*, *sugE(c)*, *mdfA*, *ydgE*/*ydgF*, *qac* and *sugE(p)*) conferring resistance to biocides, such as quaternary ammonium compounds (QACs). Among isolates from pig slaughterhouses, its abundance was lower at 33.3% (12/36).

## 4. Discussion

The study provides evidence on the diversity of antimicrobial resistance, genetic lineages, virulence factors of MDR, and extraintestinal pathogenic *E. coli* from process waters and wastewater isolated from German poultry and pig slaughterhouses.

Although aminoglycosides were not used for selective isolation of the investigated target bacteria, ARGs coding for their resistance was detected in almost all isolates, which is in contrast to results from routine resistance monitoring in Germany [28,29]. Aminoglycosides are veterinary critically important antimicrobials (VCIA), with specifically neomycin, dihydrostreptomycin and spectinomycin being very important for the treatment of septicemia as well as digestive, respiratory and urinary diseases in livestock [30]. In the human sector, aminoglycosides have been assigned to critically important antimicrobials (CIAs) due to their relevance for treating MDR Gram-negative bacteria [31]. Especially gentamicin, amikacin and tobramycin are of high importance as they are used in combination with β-lactams against emerging MDR *Acinetobacter*, *Pseudomonas* and *Enterobacter* species [32].

Although the general use of antimicrobials has decreased in recent years, consumption of aminoglycosides in the German veterinary sector has slightly increased [1]. Interestingly, although the highest amount of aminoglycosides was used in fattening chickens in Germany, the abundance of ARGs coding for aminoglycoside resistance in isolates from pig slaughterhouses was comparably high as in poultry isolates. This might be due to co-selection through other antimicrobials that are often used in pigs, e.g., macrolides, lincosamides and tetracyclines [1] or linked with our selective isolation procedure. However, the occurrence of *aac(6’)-Ib-cr5* in isolates from pig slaughterhouses is worrying, as the AAC(6’)-Ib-cr5 enzyme confers reduced susceptibility to quinolones and amikacin [33] and is increasingly reported in human isolates of the genus *Acinetobacter* as well as the *Pseudomonadaceae* family. Both genera comprise species assigned to the ESKAPE bacteria, which cause the majority of hospital infections with antibiotic-resistant bacteria in the European Union (EU) and USA [34,35].

All isolates harbouring *aac(6’)-Ib-cr5* also carried *bla*_OXA-1_. This finding is in line with Livermore and colleagues (2019) [9], who found that resistance to piperacillin-tazobactam is often associated with *bla*_OXA-1_ that encodes a penicillinase with weak affinity for inhibitors such as tazobactam or clavulanate and is commonly associated with co-carriage of *aac(6’)-Ib-cr.* Since all piperacillin-tazobactam-resistant *E. coli* carried *bla*_TEM-1_, its hyperproduction might be linked to this phenotype [8,36].

Interestingly, *E. coli* isolates carrying *aac(6’)-Ib-cr5* and *bla*_OXA-1_ belong to sequence type ST410 reported as an extraintestinal pathogen worldwide, and dissemination was described between humans companions animals, wildlife, livestock, and the environment. Furthermore, this sequence type was also associated with the emergence and/or co-occurrence of carbapenemase genes like *bla*_NDM-5_ and/or *bla*_OXA-181_ in human isolates [37]. Fortunately, the isolates of this study, as well as those described in other animal populations, still lack carbapenemase genes. However, *bla*_CTX-M-1_ and *bla*_CTX-M-15,_ as well as *bla*_CTX-M-32_, *bla*_CTX-M-14_ and *bla*_CTX-M-55_ that are often associated with ESBL-producing isolates from hospitals and ambulatory patients in Germany, were detected in isolates from both poultry and pig slaughterhouses [38].

Macrolides belong to the “highest priority critically important antimicrobials” (HPCIA) in human medicine. However, besides tetracyclines and penicillins, they belong to the most commonly used antimicrobials in fattening pigs in Germany [39]. The occurrence of resistance genes in almost all tested isolates is worrying since it is not excluded that the corresponding genes are located on the same plasmid as ARGs coding for resistance to β-lactam and aminoglycosides. Thus, macrolides in veterinary medicine may contribute to the co-selection and spread of resistance to CIAs for humans. Furthermore, *erm* genes could be transferred to Gram-positive pathogens (e.g., enterococci, streptococci, staphylococci) and result in MLS_B_ (macrolide, lincosamide, streptogramin B) cross-resistance [40], compromising the efficacy of HPCIA for humans such as erythromycin macrolides and clindamycin.

As a notifiable number of isolates carried QAC genes, the use of QACs in the food industry, in particular in slaughterhouses, may provide additional selection pressure for clinically relevant *E. coli* with acquired resistance to other antimicrobial classes [41].

It is worrying that most detected STs in our study have also been reported in human infections worldwide [11,38]. ST10, ST58, ST88, ST117, ST131, ST167, ST410, ST617 and ST648 belong to the most widely disseminated extraintestinal pathogenic strains worldwide, causing infections of the bloodstream, urinary (UTI) and respiratory tracts as well as meningitis and necrotizing enterocolitis [11]. Furthermore, clones of ST34, ST101, ST155, ST361, ST744, ST1011, ST1170, ST1284 and ST1431 have been isolated in Germany from nosocomial and ambulant (UTIs) infections [38]. Of note, these clones were detected at all tested sampling sites along the slaughtering process, including scalding water (ST10, ST58, ST361) and the effluents of in-house WWTPs (ST10, ST117, ST410, ST648). *E. coli* allocated to STs detected in scalding water (ST10, ST58, ST361) have also been reported in chicken meat in different countries worldwide [42,43,44]. This emphasizes the potential role of polluted process waters for cross-contamination of carcasses and raw meat. Furthermore, our results show that in-house WWTPs of poultry and pig slaughterhouses may also play a relevant role in disseminating *E. coli* with zoonotic potential into the environment, including surface waters. *E. coli* ST10, ST117, ST410, ST648 have already been detected in surface waters in different European countries such as the Netherlands [45], Norway [46] and Switzerland [47], but it has to be mentioned that the sources of this contamination may have had multiple origins.

It is important that a significant percentage of *E. coli* isolates, especially from poultry slaughterhouses, belonged to the ExPEC pathotype that is known for its potential to cause human disease [48]. Such isolates were detected in scalding water and process water from eviscerators. As a consequence, cross-contamination of carcasses and their introduction into the food chain cannot be ruled out. *Escherichia coli* exhibiting an ExPEC pathotype have already been detected in a variety of different food products, including retail poultry meat [14,49,50] and pork [51]. Some ExPEC isolates from animals have been shown to possess similar virulence gene profiles as human-associated ExPEC [52]. The mannose-binding type 1 pilus tip protein FimH plays a role in the invasion of ExPEC strains and their translocation from the intestine that can cause gut-derived bacteremia and sepsis [53]. Furthermore, FimH plays a vital role in lower UTI and kidney infections [54]. *AstA* that was detected in a notifiable number of recovered isolates, especially from poultry slaughterhouses, encodes the enteroaggregative *E. coli* heat-stable enterotoxin and might confer the ability to produce diarrhea [55]. Such isolates have already been associated with a waterborne outbreak of diarrhea [56]. Siderophores enable the acquisition and use of essential iron (Fe^2+^/Fe^3+^) by bacteria and are essential for survival in the host [57]. *KpsM II* encodes a group 2 capsule known as a protection factor against phagocytosis [57]. Given that requirements for colonization of different physiological compartments often overlap, the incoming ExPEC isolates might be able to establish residence in the intestinal tract of the new host and cause disease afterward [58]. Because of the potential of these isolates to cause UTI in humans, these infections have been referred to as foodborne UTI [52].

Of special concern is the occurrence of UPEC strains in effluents of in-house WWTPs of poultry slaughterhouses since UPEC are the major cause of UTIs, accounting for 75% of infection cases [59]. Zhi and colleagues (2020) suggested that UPEC of human origin appear to be specifically adapted to survive wastewater treatment processes such as chlorination, UV irradiation and activated sludge [60]. This elevates the concerns for public health since inadequate treatment of wastewater contributes to the establishment and persistence of environmental reservoirs of UPEC, facilitating their circulation among different populations.

Of particular importance is the high abundance of genes conferring arsenic resistance. Zhang and colleagues (2020) reported on the shift of antibiotic resistance genes (ARGs) and mobile genetic elements in bacteria from different surface waters (i.e., rivers, lakes, and reservoirs) due to arsenic pollution [61]. Furthermore, resistances to certain antimicrobials (e.g., enrofloxacin) are reported to be co-selected by other heavy metals, including copper, zinc, mercury, silver and nickel [62,63]. Heavy metals can reach high concentrations in different environmental settings, including agricultural production, and remain stable for prolonged periods of time [64], playing a notifiable role in the spread and proliferation of ARGs.

## 5. Conclusions

Occurrence of ESBL-producing, MDR *E. coli* with ExPEC, and UPEC pathotypes in process waters along poultry and pig slaughtering chains pose an elevated risk of employees’ exposure to contaminated waters. The resistance patterns of the isolates among poultry or pig slaughterhouses were only slightly different. However, there were some considerable differences between isolates from the individual poultry and pig slaughterhouses, e.g., higher resistance rates to CIP, LVX and TZP among poultry isolates compared to those recovered from pig slaughterhouses. Furthermore, such clones of clinical relevance may be introduced into the food chain through cross-contamination of carcasses during scalding. Moreover, the inadequate treatment of the polluted wastewater in in-house WWTPs of poultry and pig slaughterhouses may result in disseminating antibiotic-resistant clinically relevant bacteria into the environment, e.g., receiving water bodies. Consequently, a contribution of these process waters, similarly to all effluents of WWTPs to a possible spread of bacteria into the general population, cannot be ruled out.

## Figures and Tables

**Table 1:** Antibiotic resistance genes and their combinations detected in selected *E. coli* isolates recovered from wastewater and process waters from poultry and pig slaughterhouses.

	Poultry Slaughterhouses, *n* = 35		Pig Slaughterhouses, *n* = 36	
	Genes	Percentage	Genes	Percentage
**Aminoglycosides**	*aadA1, aadA2*	22.9	*aadA1*	8.3
	*aadA2*	14.3	*aadA1, aadA2*	8.3
	*aadA1, aph(3″)-Ib, aph(6)-Id*	11.4	*aac(3)-IIe, aph(3″)-Ib, aph(6)-Id*	5.6
	*aadA22*	11.4	*aadA1, aadA2, aph(3″)-Ib, aph(6)-Id*	5.6
	*aac(3)-IIe, aadA1*	5.7	*aadA1, aph(3″)-Ib, aph(6)-Id*	5.6
	*aadA, aadA5, aph(3″)-Ib, aph(6)-Id*	5.7	*aac(3)-IId, aph(3″)-Ib, aph(3′)-Ia, aph(6)-Id*	2.8
	*aac(3)-IId, aadA2*	2.9	*aac(3)-IIe, aadA1*	2.8
	*aadA1, aadA2, aph(3″)-Ib, aph(3′)-Ia, aph(6)-Id*	2.9	*aac(3)-IIe, aadA1, aph(3′)-Ia*	2.8
	*aadA1, aadA5, aph(3″)-Ib, aph(6)-Id*	2.9	*aac(3)-IIe, aadA5, aph(3″)-Ib, aph(6)-Id*	2.8
	*aadA2, aph(3″)-Ib, aph(6)-Id*	2.9	*aac(3)-IIe, aph(3″)-Ib, aph(6)-Id*	2.8
	*aadA22, aph(3″)-Ib;aph(3′)-Ia, aph(6)-Id*	2.9	*aac(3)-IVa, aadA1, aph(3″)-Ib, aph(3′)-Ia, aph(4)-Ia, aph(6)-Id*	2.8
	*aadA5, aph(3″)-Ib, aph(6)-Id*	2.9	*aadA1, aadA5*	2.8
			*aadA1, aadA5, aph(3″)-Ib, aph(3′)-Ia, aph(6)-Id*	2.8
			*aadA5*	2.8
			*aadA5, aph(3″)-Ib, aph(6)-Id*	2.8
			*aph(3″)-Ib, aph(3′)-Ia, aph(6)-Id*	2.8
			*aph(3″)-Ib, aph(6)-Id*	2.8
	**Overall**	**88.6**		**66.7**
**Aminoglycosides and Fluoroquinolones**			*aac(6’)-Ib-cr5*	16.7
	**Overall**			**16.7**
**β-lactams**	*bla*_EC_, *bla*_SHV-12_	20.0	*bla*_CTX-M-1_, *bla*_EC_	25.0
	*bla*_EC_, *bla*_TEM-52_	17.1	*bla*_CTX-M-1_, *bla*_EC_, *bla*_TEM-1_	22.2
	*bla*_EC_, *bla*_TEM-1_	11.4	*bla*_CTX-M-15_, *bla*_EC_, *bla*_OXA-1_	13.9
	*bla*_CTX-M-1_, *bla*_EC_, *bla*_TEM-1_	8.6	*bla*_CTX-M-55_, *bla*_EC_	8.3
	*bla*_CTX-M-32_, *bla*_EC_, *bla*_TEM-1_	8.6	*bla*_EC_, *bla*_OXA-1_, *bla*_TEM-1_	8.3
	*bla*_EC_, *bla*_SHV-12_, *bla*_TEM-1_	8.6	*bla*_CTX-M-15_, *bla*_EC_, *bla*_TEM-1_	5.6
	*bla*_CTX-M-1_, *bla*_EC_	5.7	*bla*_EC_, *bla*_SHV-12_	5.6
	*bla*_CTX-M-15_, *bla*_EC_, *bla*_TEM-1_	5.7	*bla*_CTX-M-1_, *bla*_EC_, *bla*_OXA-1_	2.8
	*bla*_EC_, *bla*_SHV-2_, *bla*_TEM-1_	5.7	*bla*_CTX-M-137_, *bla*_EC_, *bla*_TEM-1_	2.8
	*bla*_ADC_, *bla*_OXA_	2.9	*bla*_CTX-M-14_, *bla*_EC_, *bla*_TEM-1_	2.8
	*bla*_CTX-M-1_, *bla*_EC_, *bla*_SHV-106_, *bla*_TEM-1_	2.9	*bla*_CTX-M-15_, *bla*_EC_, *bla*_OXA-1_, *bla*_TEM-1_	2.8
	*bla*_EC_, *bla*_TEM-1_, *bla*_TEM-19_	2.9		
	**Overall**	**100**		**100**
**Phenicols**	*cmlA1*	17.1	*catB3*	13.9
	*catA1*	11.4	*floR*	11.1
	*catA1, cmlA1*	5.7	*catA1, floR*	8.3
			*cmlA1, floR*	8.3
			*catB3, cmlA1*	2.8
			*cmlA1*	2.8
	**Overall**	**34.3**		**47.2**
**Diaminopyrimidines**	*dfrA1*	14.3	*dfrA17*	13.9
	*dfrA12*	8.6	*dfrA12, dfrA36*	8.3
	*dfrA17*	5.7	*dfrA1*	2.8
	*dfrA1, dfrA12*	2.9	*dfrA1, dfrA17*	2.8
	*dfrA1, dfrA14*	2.9	*dfrA12*	2.8
	*dfrA1, dfrA17*	2.9	*dfrA12, dfrA5*	2.8
	*dfrA12, dfrA17*	2.9	*dfrA14*	2.8
	*dfrA14*	2.9	*dfrA17, dfrA7*	2.8
	*dfrA5*	2.9	*dfrA5*	2.8
	**Overall**	**45.7**		**41.7**
**Macrolides**	*emrD*	77.1	*emrD*	61.1
	*emrD, mph(B)*	8.6	*emrD, mph(A)*	30.6
	*emrD, mph(A)*	5.7	*emrD, erm(B)*	2.8
	*emrD, erm(B)*	2.9	*emrD, mph(A), mph(B)*	2.8
	*emrD, mef(B)*	2.9	*emrD, mphE, mef(B),msrE*	2.8
	**Overall**	**97.1**		**100**
**Lincosamides**	*lnu(F)*	48.6	*lnu(G)*	11.1
	*lnu(F), lnu(G)*	2.9	*lnu(F)*	2.8
	**Overall**	**51.4**		**13.9**
**Phosphonic Acid (Fosfomycin)**	*fosA*	2.9		
	*abaF*	2.9		
	**Overall**	**5.8**		
**Tetracyclines**	*tet(A)*	48.6	*tet(A)*	36.1
	*tet(B)*	8.6	*tet(B)*	13.9
	*tet(A), tet(M)*	2.9	*tet(A), tet(M)*	8.3
			*tet(A), tet(B)*	2.8
	**Overall**	**60.0**		**61.1**
**Sulfonamides**	*sul2*	28.6	*sul2*	25.0
	*sul1, sul2*	14.3	*sul1, sul2, sul3*	11.1
	*sul3*	14.3	*sul1*	8.3
	*sul1*	5.7	*sul1, sul2*	8.3
	*sul2, sul3*	5.7	*sul3*	8.3
	*sul1, sul3*	2.9	*sul2, sul3*	2.8
	**Overall**	**71.4**		**63.9**
**Streptothricin (Nucleosides)**	*sat2*	5.7	*sat2*	8.3
	**Overall**	**5.7**		**8.3**
**Fluoroquinolones**	*qnrS1*	17.1	*qnrS1*	11.1
	*qnrB19*	11.4	*qnrB19*	5.6
	*oqxA, oqxB*	2.9		
	**Overall**	**31.4**		**16.7**
**Cyclic Polypeptides (Colistin)**	*mcr-1.1*	11.4	*mcr-1.1*	8.3
	**Overall**	**11.4**		**8.3**

**Table 2:** Multi-locus sequence types (MLST) distribution of selected *E. coli* isolates recovered from poultry and pig slaughterhouses.

*E. coli, n* = 35 Poultry Slaughterhouses			*E. coli, n* = 36 Pig Slaughterhouses		
Sequence Type	*n*	%	Sequence Type	*n*	%
ST117	3	8.6	ST410	5	13.9
ST10	2	5.7	ST10	4	11.1
ST224	2	5.7	ST359	3	8.3
ST533	2	5.7	ST744	3	8.3
ST648	2	5.7	ST58	2	5.6
ST1011	2	5.7	ST88	2	5.6
ST1730	2	5.7	ST117	2	5.6
ST3995	2	5.7	ST101	1	2.8
ST4994	2	5.7	ST131	1	2.8
ST34	1	2.9	ST156	1	2.8
ST58	1	2.9	ST167	1	2.8
ST101	1	2.9	ST224	1	2.8
ST135	1	2.9	ST398	1	2.8
ST155	1	2.9	ST542	1	2.8
ST162	1	2.9	ST617	1	2.8
ST297	1	2.9	ST641	1	2.8
ST361	1	2.9	ST1170	1	2.8
ST457	1	2.9	ST1284	1	2.8
ST515	1	2.9	ST1431	1	2.8
ST711	1	2.9	ST3595	1	2.8
ST1485	1	2.9			
ST6617	1	2.9			
unknown STs	3	8.6	unknown STs	2	5.6

**Table 3:** Virulence factors detected in selected *E. coli* isolates recovered from wastewater and process waters from poultry and pig slaughterhouses.

		*E. coli* (*n* = 35), % Poultry Slaughterhouses	*E. coli* (*n* = 36), % Pig Slaughterhouses
**Adhesins**			
*fimH*	Type 1 fimbriae	94.3	91.7
*papC*	Genes of P fimbriae operon	5.7	11.1
*papEFG*	Genes of P fimbriae operon	5.7	8.3
*sfa/foc*	S or F1C fimbriae	5.7	11.1
*focG*	F1C fimbriae adhesin	0	0
*iha*	Adhesin siderophore	0	0
*F10 papA*	P fimbriae subunit variant	0	0
*tsh*	Temperature sensitive hemagglutinin	0	0
*hra*	Heat-resistant agglutinin	0	0
*afa/draBC*	Dr-binding adhesins	0	0
**Toxins**			
*astA*	Enteroaggregative *E. coli* toxin	40.0	16.7
*vat*	Vacuolating toxin	8.6	5.6
*pic*	Serine protease	8.6	2.8
*hlyD*	Alpha-hemolysin	0	2.8
*cnf1*	Cytotoxic necrotizing factor	0	2.8
*sat*	Secreted autotransporter toxin	0	0
**Siderophores**			
*iutA*	Aerobactin receptor	51.4	58.3
*iroN*	Salmochelin receptor	48.6	30.6
*fyuA*	Yersiniabactin receptor	37.1	27.8
*ireA*	Siderophore receptor	0	0
**Capsule**			
*kpsM II*	*kpsM II* group 2 capsule	22.9	5.6
*K1*	K1 group 2 capsule variants	0	0
*K2*	K2 group 2 capsule variants	0	0
*K5*	K5 group 2 capsule variants	0	0
*kpsMT III*	Group 3 capsule	0	0

**Table 4:** Heavy metal resistance genes detected in *E. coli* isolates recovered from wastewater and process waters from poultry and pig slaughterhouses.

Metal	*E. coli* (*n* = 35), % Poultry Slaughterhouses	*E. coli* (*n* = 36), % Pig Slaughterhouses
Arsenic	97.1	100.0
Copper	5.7	13.9
Copper/silver	5.7	27.8
Mercury	25.7	38.9
Silver	5.7	27.8
Tellurium	2.9	2.8
Nickel	2.9	0

## Data Availability

The data for this study were deposited in the Sequence Read Archive (SRA) at NCBI under accession number PRJNA706398.

## Supplementary Materials

The following are available online at https://www.mdpi.com/article/10.3390/microorganisms9040698/s1, Table S1: Selected characteristics of the investigated slaughterhouses; Table S2: Number of samples taken at each sampling point in poultry slaughterhouses; Table S3: Number of samples taken at each sampling point in pig slaughterhouses; Table S4: Isolation sites of the individual isolates and their accession numbers.
