# Supplementary material for: Clinically Relevant Escherichia coli Isolates from Process Waters and Wastewater of Poultry and Pig Slaughterhouses in Germany
Source: Microorganisms. 2021 Mar 28;9(4):698. doi: 10.3390/microorganisms9040698 (PMC8066038; doi:10.3390/microorganisms9040698)
Supplement: Supplementary file 1 [file microorganisms-09-00698-s001.zip › microorganisms-1156761-suppl-for conversion/Supplementary Material, Tables S1-S3.pdf]

Supplementary Material

Clinically relevant *Escherichia coli* isolates from process waters and wastewater of poultry and pig slaughterhouses in Germany

Mykhailo Savin, Gabriele Bierbaum, Judith Kreyenschmidt, Ricarda Maria Schmithausen, Esther Sib, Silvia Schmoger, Annemarie Käsbohrer and Jens Andre Hammerl

Corresponding authors:

Dr. Mykhailo Savin, Institute for Hygiene and Public Health, Medical Faculty, University of Bonn, Campus Venusberg 63, 53127 Bonn, Germany

E-mail: m.savin@uni-bonn.de

Dr. Jens Andre Hammerl, Unit Epidemiology, Zoonoses and Antimicrobial Resistances, Department of Biological Safety, German Federal Institute for Risk Assessment, Max-Dohrn Str. 8-10, D-10589 Berlin, Germany

E-mail: jens-andre.hammerl@bfr.bund.de

Table S1: Selected characteristics of the investigated slaughterhouses.

	Poultry Slaughterhouse S1	Poultry Slaughterhouse S2	Pig Slaughterhouse S1	Pig Slaughterhouse S2
Daily slaughtering capacity	>100,000	>100,000	>3,000	>3,000
Daily amount of wastewater, m^3	600	3,600	2,100	550
Type of WWTP	Membrane bioreactor (MBR) with immersed ultrafiltration membranes	Conventional biological WWTP	Conventional biological and physical-chemical WWTPs	Conventional biological WWTP
Direct discharger	Yes	Yes	No	No

Table S2: Number of samples taken at each sampling point in poultry slaughterhouses.

	**Poultry slaughterhouses S1/S2[a, b]**
Poultry Transport Trucks	5
Poultry Transport Crates	10
Stunning Facilities	10
Scalding water	10
Eviscerators	10
Production Facilities	5
Influent in-house WWTP[c, d]	16
Effluent in-house WWTP[c, d]	16

a – sampling campaigns in December 2016, July 2017, August 2017, December 2017, February 2018

b – sampling campaigns in October 2017, November 2017, March 2018, April 2018, Mai 2018

c – additional sampling campaigns in the S1 in-house WWTP in September 2017, November 2017, March 2018

d – additional sampling campaigns in the S2 in-house WWTP in July 2018, September 2018, October 2018

Table S3: Number of samples taken at each sampling point in pig slaughterhouses.

	Pig slaughterhouses S1[a]/S2[b]	
Wastewater from animal transporters	2/10	1/10
Wastewater from holding pens	2/7	0/7
Scalding and dehairing water	2/10	0/10
Aggregate wastewater from producing facilities	5/10	0/10
Influent biological WWTPs	7/10	0/10
Influent chemical-physical WWTP	4/5	0/5
Effluent biological WWTPs	4/10	0/10
Effluent chemical-physical WWTP	0/5	0/5

a – sampling campaigns in S1: March 2017, September 2017, October 2017, January 2018, February 2018

b – sampling campaigns in S2: March 2018, April 2018, May 2018, June 2018, July 2018.
